# Determinants of Community Participation in Collective Management of Natural Resources in Sub-Saharan Africa: A Systematic Review

**DOI:** 10.1007/s00267-026-02568-0

**Published:** 2026-08-04

**Authors:** Alexander Omondi Imbo, Uta Wehn, Kenneth Irvine

**Affiliations:** 1 IHE Delft Institute for Water Education, Delft, The Netherlands; 2 Centre for Science and Technology Studies, Leiden University, Leiden, The Netherlands; 3 Aquatic Ecology and Water Quality Management, Wageningen University, Wageningen, the Netherlands

**Keywords:** Community participation, Community-based natural resources management, Collective action, Determinants, Drivers and barriers, Incentives and disincentives

## Abstract

Community participation in collective management of environmental and natural resources is increasingly of interest among the sustainable development circles and scholarly discourse. There is theoretical consensus that sustainable management of natural resources is highly reliant on effective local community participation. In practice, however, realizing broad and active participation of local communities in a manner that is economically, socially and ecologically sustainable remains inconsistent. While extensive case study research has examined the dynamics of community participation aimed at understanding the enabling factors, there remains a notable gap in systematic analyses that synthesize recurring themes across these studies. In this research, we draw on the Institutional Analysis and Development Framework as an organizing theoretical framework, to review the empirical literature on collective management of common-pool natural resources in sub-Saharan Africa, in order to collate knowledge for a clearer understanding of the underlying determinants of community participation. The findings show that: the factors influencing community participation are complex, multidimensional and intersecting; different natural resources offer different combinations of ecological, economic, and social incentives that condition perceptions of benefits and costs that drive community participation; local communities are not homogenous, and the preferences and responses of individual members depend on their distinct identities and social settings; and sound local governance is the cornerstone of community participation. These insights result in specific, locally relevant policy directions for effective community participation.

## Introduction

Since the 1980s, governance reforms in developing countries have led to a proliferation of community-based models of environmental and natural resource management, a trend that has been accelerated during the last decade by the global quest for sustainable development. In these models, local communities are empowered to exercise certain rights to manage common pool natural resources for local benefit, often in partnership with other actors such as government and non-governmental organisations (Roe and Nelson 2009). An abundance of terms are used in the literature to describe the variants of these management approaches depending on the socio-political and ecological contexts. These include Community-based Conservation (CBC), Community-based Natural Resource Management (CBNRM), Integrated Conservation and Development Projects (ICDPs), Participatory Management (PM), and Collaborative Management (CM) (Child and Murphree 2004; King 2010; Roe 2011; Whande 2007). The approaches and their variants give substantive or limited property rights over natural resources to local communities, place different emphasis on commercial or subsistence uses, depend on consumptive or non-consumptive forms of resource utilisation, and can be formal or informal, but often straddling both (Chevallier 2016; Cockerill and Hagerman 2020; van Wijk et al. 2015). They may be driven by local communities at the grassroots through self-mobilisation or, alternatively, initiated by external agents such as government and non-governmental organisations in a top-down manner (Barrow and Murphree 2001; Chuenpagdee and Jentoft 2007; Katikiro et al. 2024; Mahajan et al. 2021). The approaches require devolution of some decision-making power and, therefore, entail the development of existing or crafting of new institutions and organisations for local-level management (Armitage 2005).

The rationale for involving local communities in the management of natural resources has been clearly articulated. A strongly held tenet is that local communities are willing and able to sustainably conserve natural resources if they are involved in their management and receive tangible benefits in return (Campbell and Vainio-Mattila 2003; Scanlon and Kull 2009; Songorwa 1999). Community participation is also supported on grounds of poverty alleviation through enhanced access to material benefits, as well as inclusion in decision-making which engenders empowerment, democracy and environmental justice (Campbell and Vainio-Mattila 2003; Foyet 2024; Meinzen-Dick and Knox 1999). Furthermore, community participation reduces the operational costs of conservation (Hutton et al. 2005). Generally, community-based approaches are premised on the expectation of managing natural resources to concurrently address economic development, social empowerment, and environmental conservation goals (Binot et al. 2009; Murphree 2009). Supported by such powerful narratives, the approaches have gained currency as prominent rural development strategies in many countries in the region across different resource regimes (Abensperg-Traun 2009; Armitage 2005; Blaikie 2006; Nelson et al. 2021; Wily 2002).

However, evidence from empirical research shows that community-based approaches have so far yielded mixed outcomes (successes and failures) with many interventions falling short of their ecological, social and economic expectations (Blaikie 2006; Brooks et al. 2013; Dressler et al. 2010; Galvin et al. 2018; Heffernan 2022; Kellert et al. 2000; Mahajan et al. 2021; Measham and Lumbasi 2013; Milupi et al. 2017; Nelson et al. 2021; Nunan et al. 2020; Roe et al. 2013; Salerno et al. 2021; Shackleton et al. 2002). One attributing factor is that, during implementation, it remains difficult to ensure active participation of the majority of community members during all stages of resource management from decision-making to sharing of benefits, which is a requisite for such approaches to succeed (Agarwal 2001; Barrow and Murphree 2001; Calfucura, 2018; Kamoto et al. 2013; Keane et al. 2016; Miller et al. 2024; Milupi et al. 2017; Stone and Nyaupane 2014; Thakadu 2005; Vermeulen and Sheil 2007). In reality, many initiatives tend to exclude significant segments of the targeted communities, such as the marginalised, and are often marked by intermittent co-operation with collective decisions for resource use. For instance, gender biases embedded in community norms and expectations frequently hinder women’s involvement in decision-making in collective natural resources management (Cassidy 2001; Keane et al. 2016; Khumalo and Freimund 2013; Sommerville et al. 2022). Due to various reasons, local resource users commonly fail to comply with resource use regulations which is required for the initiatives to remain viable (e.g. Bedelian and Ogutu 2017; Etiegni et al. 2017; Imbo et al. 2026). Thus it is argued that assumptions regarding community participation are often oversimplified (Balint and Mashinya 2006; Li 2002), or that local communities are no longer harmonious or environmentally friendly owing to social changes (Wilshusen et al. 2002). The participation conundrum highlights the need to take better account of the complex social-ecological contextual factors underlying community participation in natural resources management. Although there is substantial empirical research on community participation dynamics and the complexities of collective management of natural resources, systematic comparative evaluations that collate and synthesise such evidence remain limited (e.g. Brooks et al. 2013; Galvin et al. 2018; Waylen et al. 2010). Such a synthesis of evidence is necessary, however, to inform community participation interventions that can effectively address both environmental conservation and human welfare needs.

It has been suggested in the literature that promoting community participation in conservation is about understanding what motivates or restricts actors (landholders and resource users) in taking certain actions (resource use decisions) (Moon et al. 2012). For this study, we conducted a systematic review of published scholarly literature to assess the drivers and barriers to, and the incentives and disincentives for community participation in collective management of common-pool natural resources. The study aimed to generate an integrated overview for learning across disparate contexts. The study concentrated on sub-Saharan Africa because the region has been a major laboratory for community-based approaches that has played a key role in their global spread (Nelson et al. 2021). Whereas community participation is nested in broader socio-political environments (Berkes 2007), this study focused on local contextual factors and conditions because local factors have direct or immediate influence on the strategies of resource users and can often be influenced by the community of resource users (Edwards and Steins 1999). Macro-level mechanisms are an important consideration, however understanding the influence of micro-level mechanisms better illustrate how local people are incentivised to participate (Silva and Mosimane 2014). The study proposed to answer the following research question: What are the underlying determinants of community participation and co-operation in collective management of common-pool natural resources? Effective participation is defined in terms of the breadth and depth of community engagement, i.e. the willing and active participation of the majority of community members (breadth) during all stages of resource management including decision-making, implementation of activities, and benefits access (depth) (Cornwall 2008). The remainder of the paper is organised as follows. Section “Organising Framework” outlines the conceptual framework underpinning the study. Section “Methods” elaborates the approach used to conduct the systematic review. Section “ Results” presents the results of the systematic review. Section “Discussion” discusses the relevance of the findings and their policy implications. Finally, Section “Conclusion” draws the conclusions of the study.

## Organising Framework

**Fig. 1:**
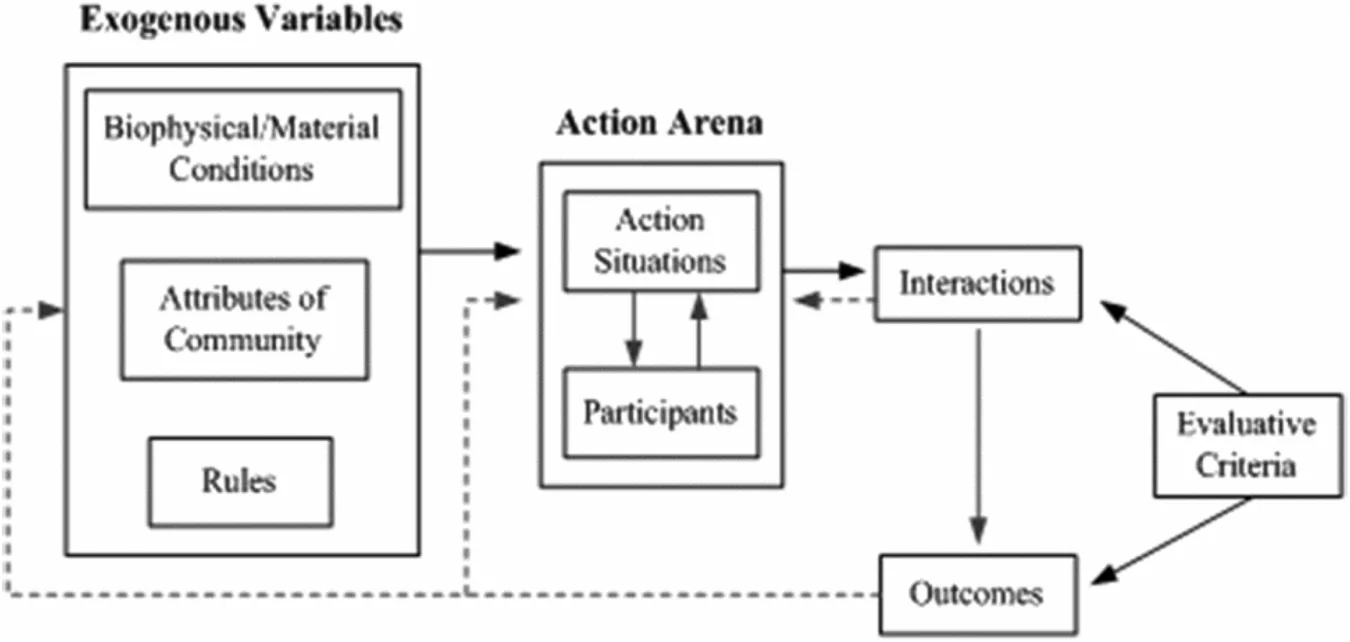
The IAD framework (Source: Ostrom, 2005)

The Institutional Analysis and Development framework or IAD (Ostrom 2005), depicted in Figure 1, was used to structure the review of factors that shape local community members’ participation in conservation. The IAD is a well-established framework for analysing how institutions and other contextual factors influence people’s strategic decisions for resource management. The framework has been broadly tested in a wide array of empirical settings (Cole et al. 2019; Ostrom 2005), affirming that it is particularly suitable for application to common pool resources that require co-operation for sustainable management. In its basic form, the IAD framework pays attention to the space for participation, referred to as the action arena, which is inclusive of a specific resource management activity referred to as the action situation, and the people who participate, referred to as the actors. The action arena, which is the focal point of analysis, is influenced by exogenous variables (contextual factors) which are environmental, societal and institutional in nature, which provide opportunities and incentives for actors to behave in particular ways. Actors’ decisions depend on their characteristics and capabilities as well as the incentives (costs and benefits) they perceive for various behaviours (Ostrom 2005). The assumption is that actors will adopt strategies to maximise their self-interests within the rules (Thomson and Freudenberger 1997). The behaviours of actors are stipulated to accrue into predictable patterns of interaction (e.g. co-operation or non-cooperation) which produce certain outcomes (economic, social and environmental). The interactions and outcomes may feed back into and reconstitute the system, i.e. influence future behaviours (Ostrom 2005).

Applying the IAD as an organising framework, the study sought to understand the drivers and barriers that people face and their sources, which are related to: (i) individual characteristics and capabilities (ii) biophysical characteristics; (iii) attributes of the community; (iv) rules in use; (v) patterns of interaction (process factors); (vi) and perceived outcomes. It was hypothesized that local people will be motivated (willing and able) to participate and co-operate in conservation if perceptions of benefits outweigh perceptions of the costs of participating, e.g. the costs of relinquishing alternative behaviours (such as loss of livelihoods) (Ostrom 1990). Accordingly, local people are predicated to alter their land use and resource management decisions and comply with environmental conservation decisions and policies to promote their best self-interests in terms of tangible and intangible benefits.

## Methods

In order to assess the drivers and barriers to effective community participation in the management of common-pool natural resources in sub-Saharan Africa and the strategies that have been deployed to promote participation, we conducted a systematic review of accessible peer-reviewed journal literature. The review followed the PRISMA (Preferred Reporting Items for Systematic Reviews and Meta-Analyses), which entails formulating the question(s) to be addressed, formulating the selection criteria for studies, applying the selection criteria to identify studies, analysing the selected studies, and synthesising results (Page et al. 2021). To identify articles for review, we searched the Scopus and Google Scholar databases using search terms comprising of the overlapping terminologies commonly used in the literature to refer to community-based approaches to environmental and natural resource management in the context of Africa (e.g. Attwell and Cotterill 2000; Brooks et al. 2013; Child & Murphree 2004; Galvin et al. 2018; Gruber, 2010; King, 2010; Roe, 2011; Roe et al. 2009), combined with terms denoting the study objective (identifying factors that influence participation) using Boolean operators ‘OR’ and ‘AND’ (e.g. Atkinson and Cipriani 2018) (Box 1). The search was conducted in June 2025.

**Fig. 2:**
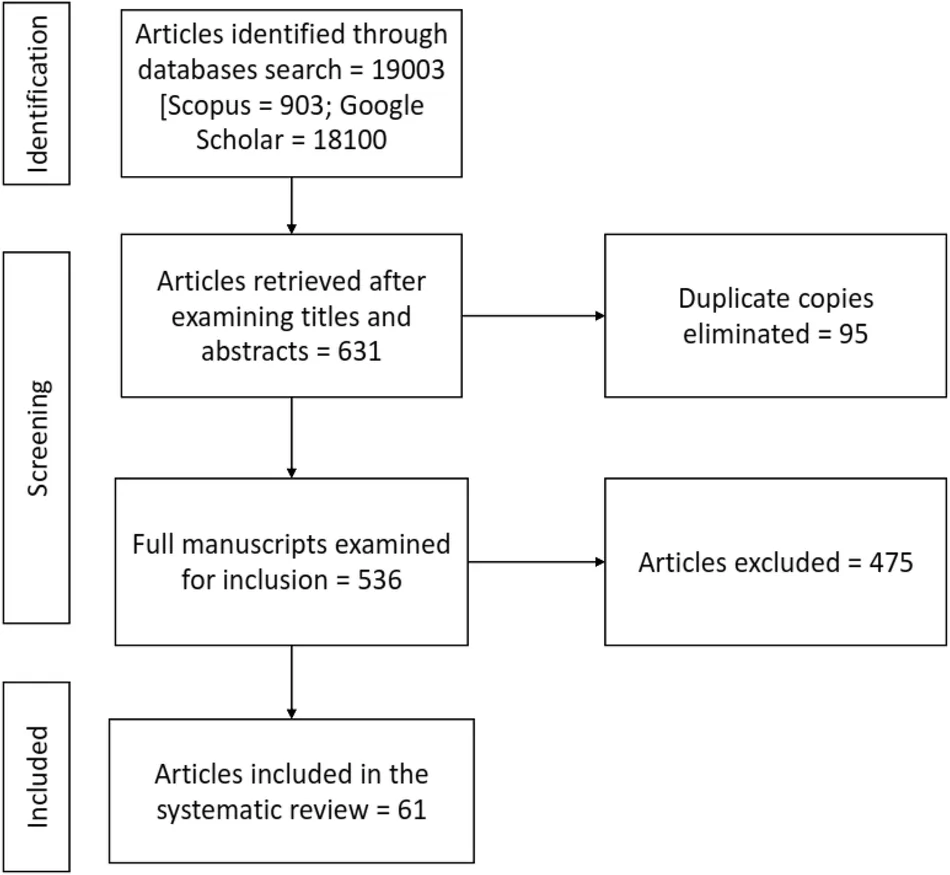
Flow-chart of search to inclusion process

Fig. 2 shows that the search yielded 19003 hits (903 in Scopus and 18100 in Google Scholar). Initial screening examined the titles and abstracts of all the articles, resulting in 631 articles that were retrieved and stored in the Endnote software. Subsequently, 95 duplicate copies were detected and eliminated. Full manuscripts of the remaining 536 articles were examined for inclusion or exclusion. Finally, 61 articles published between 1998 and 2025, were selected for inclusion in this systematic review (Supplementary Material 1). All articles that met the following inclusion criteria were incorporated in the review:

- Peer reviewed journal publication (conference papers, books, book chapters, theses and grey literature were excluded).
- Empirical case study relying mainly on primary data sources (systematic reviews and meta-analyses were excluded).
- Case study from a country in sub-Saharan Africa (case studies from other parts of the world were excluded).
- The case entails community participation in the management of environmental and natural resources, i.e. some level of involvement in decision-making, activity implementation, and/ or benefits access (studies that reported only token or passive community participation such as through protected area outreach programs were excluded).
- Reporting on factors that influence local community participation in collective management of natural resources, including perceptions and attitudes which are indicative of reasons for conservation support or lack of.

A comprehensive coding scheme was developed to extract insights from the selected articles thematically. The coding scheme was set up in MAXQDA software where all articles were organised into a single project folder, within which there were sub-folders based on categories of identified codes. The codes were identified deductively using the main constructs of the organising framework and inductively adjusted during preliminary data analysis by including some emerging narratives from the data. The contents of the selected articles were analysed qualitatively by identifying themes and patterns within the text and classifying them into the coded categories. The analysis disentangled the complex mix of factors that influence local communities’ participation and co-operation in collective management and identified some strategies that have been implemented to promote participation.

Box 1: Search-string used for Identification of Relevant Literature “community based conservation” OR “community based natural resource management” OR “integrated conservation and development projects” OR “participatory natural resource management” OR “collaborative natural resource management” AND “factor” OR “condition” OR “motivation” OR “incentive” OR “driver” OR “barrier” OR “challenge”

## Results

### Overview of the Literature

Table 1 shows that the majority of studies included in this review, which centres on Sub-Saharan Africa, focused predominantly on wildlife management, followed by forest management. Other resource types covered are marine, rangeland, fisheries, water resources, wetland, floodplain and watershed. Some articles covered more than one resource type. Table 2 shows that the articles selected for the review covered 143 different case studies (sites) spanning 16 countries in sub-Saharan Africa (out of the 48 countries in sub-Saharan Africa (Jobarteh 2025)). Some articles covered more than one country and/ or case study, whilst some case study sites were covered in more than one article. The analysis of these studies shows that community participation is influenced by multiple interwoven factors that have been categorised into those related to attributes of the individual, the natural resources under management, the local community of resource users, the rules in use, the patterns of interaction, and the perceived outcomes.

**Table 1:** Overview of resource types covered by the reviewed articles

No.	Resource type	No. of articles
1.	Wildlife	31
2.	Forest	18
3.	Marine (conservation and fisheries)	4
4.	Rangeland	3
5.	Fisheries (inland)	2
6.	Water resources	1
7.	Wetland	1
8.	Floodplain	1
9.	Watershed	1

**Table 2:** Overview of countries and case study sites covered by the reviewed articles

No.	Country	Case study sites
1.	Botswana	5
2.	Cameroon	9
3.	DR Congo	1
4.	Ethiopia	5
5.	Ghana	14
6.	Kenya	14
7.	Madagascar	21
8.	Malawi	1
9.	Mozambique	2
10.	Namibia	41
11.	Nigeria	4
12.	South Africa	1
13.	Tanzania	14
14.	Uganda	1
15.	Zambia	7
16.	Zimbabwe	3

### Attributes of Individual Community Members

The reviewed evidence highlights that individual-level characteristics (socio-economic attributes) influence overall community participation by shaping the capabilities and opportunities for individuals to participate in collective action. The primary attributes that were identified are gender, age and wealth, which are embedded in broader structural conditions that create social differentiations and configure local power relations. Additionally, individuals’ awareness and personal values impact their motivation to participate in conservation and collective action.

Case studies across Ethiopia, Ghana, Kenya, Zambia, and Namibia, especially involving wildlife and forest management, show that patriarchal norms entrenched in local communities legitimise the dominance of men in community decision-making over natural resources, while restricting women and the youth from participating inclusively or benefitting equitably. For example, in Ethiopia’s Bale Mountains, older men were more likely than women and the youth to report receiving financial benefits from controlled hunting areas managed by local communities due to their patronage over land (Abebe et al. 2020). In northern Ghana, men dominated decision-making in Community Resource Management Areas (CREMAs) because household leadership was typically male (Baddianaah and Baaweh 2021; Robinson and Sasu 2013). Similarly, in Kenya’s Maasai Mara conservancies, land ownership was primarily held by male heads of households, which excluded most women and the youth from accessing land-related conservation income (Bedelian et al. 2024; Oduor 2020). In Zambia’s Lupande Game Management Area (GMA), women’s involvement in community meetings where conservation decisions are made was restricted by cultural norms (Wainwright and Wehrmeyer 1998), whilst in Namibia’s Mashi Conservancy, women participated highly in conservancy activities such as craft sales (which aligns with their traditional activities) but not in the making of conservancy management decisions (Lendelvo et al. 2012).

A number of the case studies indicate that wealthier households tend to enjoy greater influence and access to benefits compared with poorer households owing to their social status, ability to meet participation requirements (e.g. transaction costs) and control of key resources such as land. For instance, Baker-Médard et al. (2021) found that knowledge of and participation in conservation in two marine resource management sites in Madagascar was significantly correlated with wealth, and that individuals from wealthier households were more involved in activities related to decision-making due to their higher social standing. In Kenya’s Maasai Mara, conservancy leadership was subjugated by large land owners, while the landless and land-poor individuals were either ineligible for conservancy membership or unable to contribute land for conservation (Bedelian et al. 2024). Similar trends were observed elsewhere- wealthier households captured more benefits in Tanzania’s Ambangulu Forest (Meshack et al. 2006), while poorer households bore disproportionate costs in Madagascar’s Mangabe Forest (Ward et al. 2017; Ward et al. 2018). In Ethiopia’s Dodola region, it was found that landowners with more farmland and capital assets were more likely to participate in collective tree planting and forest management (Tesfaye et al. 2012). Some women in Namibian conservancies reported that limited financial means hindered their participation in conservancy activities such as attending meetings (Lendelvo et al. 2012). Furthermore, requirement of membership fees was noted in some cases such as in the Controlled Hunting Areas of Ethiopia’s Bale Mountains (Abebe et al. 2020) and Payment for Environmental Services (PES) scheme in Madagascar’s Central Menabe region (Sommerville et al. 2010), which may be restrictive for some households.

Many examples show that beyond socio-economic attributes, individuals’ awareness and environmental values are strong motivators for participation in conservation. Local ecological knowledge and sense of environmental responsibility were key incentives for participation in marine fisheries conservation in villages in Unguja, Pemba, Kigamboni, Kibiti, Kilwa, and Mafia in Tanzania (Drury O’Neill et al. 2024; Katikiro et al. 2024). In Nigeria’s Okwangwo Division, formal education was found to be a factor influencing community members’ preferences to engage in forest management (Ezebilo 2013). In Kenya’s Arabuko-Sokoke Forest, individuals who supported conservation were more likely to acknowledge the importance of forest ecosystem services compared to those who favoured unregulated logging (Jackson and Naughton-Treves 2012). Strong environmental values also underpinned community participation in initiatives such as Namibia’s Torra Conservancy (Scanlon and Kull 2009), Ghana’s Zukpiri CREMA and Wechiau Community Hippo Sanctuary (Baker et al. 2018; Sheppard et al. 2010), and Ethiopia’s Jemma Watershed conservation (Zewde et al. 2024). Snyman (2014) observed that people living in or adjacent to conservation areas in selected case studies in the southern Africa countries of Botswana, Malawi, Namibia, South Africa, Zambia and Zimbabwe had a strong understanding and appreciation of the importance of wildlife, which translated to positive attitudes towards participation in conservation.

### Attributes of the Natural Resources

The reviewed literature indicates that the biophysical attributes of the resources under management, such as the availability (scarcity) and ecological condition of valued resources, significantly influences the incentives local resource users (community members) perceive for participating in conservation. Because rural communities are highly dependent on land and natural resources, people’s willingness and ability to participate in conservation is tied to the availability of (and uninterrupted access to) land and essential livelihood resources. The tension between livelihoods and conservation was evident in several cases such as in the Bale Mountains in Ethiopia, the Avu Lagoon CREMA in Ghana, and the Mbarang’andu, Burunge and Enduimet Wildlife Management Areas (WMAs) in Tanzania, where creation of conservation areas constrained access to land and natural resources leading to different contestations and resistance that encumbered participation (Abebe et al. 2020; Ahmed and Gasparatos 2020; Dekker et al. 2020; Kangalawe and Noe 2012; Moyo et al. 2017). Comparable concerns were observed in Botswana’s Khama Rhino Sanctuary and Namibia’s Nata Bird Sanctuary where local people expressed their disenchantment over restrictions on traditional land uses as a consequence of establishing conservation areas (Sebele 2010; Stone and Stone 2011; Stone and Rogerson 2011). In Ghana’s Akwapim-Togo Mountain Range, there was reluctance to plant trees for afforestation because of uncertainty over future land access linked to expansion of the conservation area (Baker et al. 2018). Whilst in marine conservation in Kisakasaka and Jozani-Pete in Tanzania, mangrove degradation continued due to the local communities’ overdependence on mangroves as a livelihoods resource (Rabe and Saunders 2013; Saunders et al. 2008). On a more positive note, sufficient land and ample access to natural resources enabled the residents of Jilinkon in northern Ghana to establish a conservation area without self-limiting their access to resources which enabled cooperation (Robinson and Sasu 2013).

On the other hand, scarcity of valued resources can motivate collective action to prevent overexploitation and degradation. In Tanzania’s Ambangulu Forest and Mnazi Bay-Ruvuma Estuary Marine Park, community participation was largely motivated by widespread concerns over declining forest resources and fish stocks respectively (Katikiro et al. 2015; Meshack et al. 2006). On the contrary, where some natural resources remain abundant, the incentive for collective action may diminish because individuals can independently fulfil their subsistence needs, as observed in marine conservation in Unguja and Pemba in Tanzania where non-compliance with fisheries rules by sections of the community were justified through a belief that the ecosystem’s condition had recovered (Drury O’Neill et al. 2024).

### Attributes of the Community

Findings from the reviewed case studies suggest that social cohesion (a shared sense of unity and belonging) and social capital (the networks, relationships, and values embedded within a community that enable mobilisation) are essential drivers of collective action in resource management. For instance, broad community participation in Ghana’s Zukpiri CREMA, Kenya’s Shompole, Olkiramatian, and Il’Ngwesi conservancies, and Cameroon’s Kilum-Ijim Forest were linked to aligned interests rooted in cultural homogeneity, trust and reciprocity developed through long-standing communal land practices, and the prevalence of traditional leadership which are still revered (Abbot et al. 2001; Baddianaah and Baaweh 2021; Robinson et al. 2021). Positive effects of bonding social capital (ties among community members) were also evident in collaborative forest management in Kwapanin and Kyirayaso, landscape co-management in the Akwapim-Togo Mountain Range, and biodiversity conservation at the Wechiau Community Hippo Sanctuary in Ghana (Akamani and Hall 2015; Baker et al. 2018; Sheppard et al. 2010). High participation levels in community self-help groups like farmer associations reflected strong organizational capacity in Cameroon’s Awae and Akok communities to engage in PES initiatives for forest management (Cerbu et al. 2013). Strong sense of community belonging and attachment to place in Namibia’s Torra, Uibasen and Mayuni conservancies enabled community participation in conservation even in the absence of economic incentives (Scanlon and Kull 2009; Silva and Mosimane 2014). The evolution of community trusts for CBNRM in the Okavango Delta in Botswana into key village institutions, which enhanced participation, further illustrates the transformative role of social capital in collective action (Mbaiwa 2004).

On the contrary, heterogeneities such as those related to ethnicity and culture may create different special interest groups within a community that could constrain collective action. For example, in Burunge WMA in Tanzania, the Maasai and Waarusha ethnic groups considered themselves as native to the area and the Barbaigs ethnic group as newcomers and competitors for grazing resources, and therefore resented their inclusion in the management of rangelands (Moyo et al. 2017). Whilst in the Nata area of Botswana, participation was constrained by historical legacies that have created sour inter-tribal relations between the Bangwato and Basarwa tribes (Stone and Nyaupane 2014). Similar ethnic tensions were observed in the management of the Okavango Community Trust in Botswana (Mbaiwa 2004).

### Attributes of the Rules-in-Use

The reviewed cases underscore the crucial role of both formal and informal rules in shaping collective action, as individuals make decisions based on the interplay between the two institutional types. Informal rules (unwritten rules, norms, values, traditions, and cultural practices) can either reinforce or undermine formal conservation rules depending on how well they align. For illustration, community participation in formal conservation was in some contexts driven by cultural and spiritual connections to natural resources, as seen in the Kilum-Ijim Forest in Cameroon, the sacred groves of southwest Nigeria, the Zukpiri and Kunlog CREMAs in Ghana, and the Wechiau Community Hippo Sanctuary also in Ghana (Abbot et al. 2001; Adeyanju et al. 2022; Baddianaah and Baaweh 2021; Robinson and Sasu 2013; Sheppard et al. 2010). In the same way, traditional bylaws positively affected the perceptions of the local people towards participatory natural resource management in the Jemma Watershed in Ethiopia (Zewde et al. 2024). Conversely, in Tanzania’s Enduimet WMA, pastoralist groups rejected formal grazing rules in favour of customary grazing management practices that they felt were better suited to local realities (Dekker et al. 2020). Subsistence hunting of wildlife was rife in Wuparo, Sobbe and Kwandu conservancies in Namibia despite stringent anti-poaching rules because it is considered a legitimate cultural practice and a birth-right (Lubilo and Hebinck 2019). Likewise, in Kenya’s Lake Victoria fisheries, compliance was undermined by kinship obligations that favoured social reciprocity over formal rule adherence (Etiegni et al. 2017).

Weak enforcement of formal rules due to limited institutional and organisational capacities was found to impede compliance with conservation rules. Where rule enforcement lacks certainty or severity, rule-breaking becomes rationalized, encouraging free-riding and eroding trust in collective systems (it becomes meaningless to comply if others violate rules with no consequences). The prevalence of illicit activities (unauthorised access to resources) linked to enforcement deficits were observed across Ghana’s CREMAs (e.g., Avu Lagoon, River Asuopri, and Murugu-Mognori), marine fisheries in coastal Tanzania, inland fisheries in Kenya and Zambia, Tanzania’s GMAs such as Burunge, Namibia’s conservancies such as Wuparo, Sobbe, Kwandu, and Dzoti, and mangrove conservation in coastal Tanzania (Agyare et al. 2024; Ahmed and Gasparatos 2020; Baker et al. 2018; Drury O’Neill et al. 2024; Etiegni et al. 2017; Kahler et al. 2013; Kaluma and Umar 2021; Lubilo and Hebinck 2019; Moyo et al. 2017; Rabe and Saunders 2013; Saunders et al. 2008). In contrast, experiences in Ghana’s Zukpiri CREMA and Wechiau Community Hippo Sanctuary show that consistent local monitoring of rules and imposition of commensurate fines can establish reasonable deterrence that enhances co-operation and compliance (Baddianaah and Baaweh 2021; Sheppard et al. 2010).

The perceived legitimacy of rules plays a key role in conditioning co-operation. Rules co-developed with community members are accorded greater legitimacy and therefore tend to enjoy greater acceptance, as demonstrated in marine conservation in Tanzania’s Unguja and Pemba, where joint rule-making between communities and authorities fostered broad cooperation. In comparison, resistance to fisheries regulations was more pronounced in the Mnazi Bay-Ruvuma Estuary Marine Park, where community involvement in developing rules was minimal (Drury O’Neill et al. 2024; Katikiro et al. 2015).

Property rights play a decisive role in influencing community participation by delineating who can access and benefit from natural resources, clarifying the legal rights of local communities (powers of local institutions), and establishing incentives for long-term cooperation. Secure land tenure gives resource users confidence that they will benefit from conservation efforts, thereby encouraging long-term investment in collective action. These dynamics are illustrated in case studies from Ethiopia, Ghana, Kenya, Namibia, and Tanzania (Abebe et al. 2020; Ahmed and Gasparatos 2020; Baker et al. 2018; Bedelian et al. 2024; Hoole, 2010; Kangalawe and Noe 2012; Robinson et al. 2021). For instance, unclear land tenure in Naniga-Dera and Dirre regions in Ethiopia, limited the capacity of community institutions to enforce rangeland management rules, leading to encroachment by outsiders (Robinson et al. 2021).

### Patterns of Interactions (Process Factors)

The evidence from this systematic review strongly indicates that the entrenched challenges to community participation, ranging from exclusion and inequity in decision-making and benefit-sharing to poor co-operation and lack of compliance with collective decision, are recurrently linked to weak governance arrangements (governance defined here as the institutions, organisations, processes, and relationships through which decisions are made and implemented). Weak local governance has been attributed to imbalanced representation in local management organisations (e.g. community committees), lack of clear-cut (transparent) benefit-sharing mechanisms, pre-existing social structures and norms that re-affirm social hierarchies, limited institutional and organisational capacities, inadequate government (external) support and coordination, and insufficient devolution of powers (rights) to local communities. These governance deficiencies were consistently reported across diverse contexts in Ethiopia, Ghana, Zimbabwe, Kenya, Cameroon, Zambia, Namibia, Tanzania, Uganda, Botswana, Madagascar and Mozambique, which manifested as corruption, mismanagement, poor leadership, elite capture, gender-based marginalisation, lack of equity, lack of transparency and accountability, weak enforcement of rules, and poor communication among others (Abebe et al. 2020; Agyare et al. 2024; Ahmed and Gasparatos 2020; Balint and Mashinya 2006; Bedelian et al. 2024; Cerbu et al. 2013; Dyer et al. 2014; Etiegni et al. 2017; Hoole 2010; Kachali and Loos 2025; Kaluma and Umar 2021; Kangalawe and Noe 2012; Kansky 2022; Katikiro et al. 2015; Lepp and Holland 2006; Lewis and Phiri 1998; Lubilo and Hebinck 2019; Mbaiwa 2004; Moyo et al. 2017; Oduor 2020; Rabe and Saunders 2013; Saunders et al. 2008; Sebele, 2010; Shereni and Saarinen 2021; Silva and Mosimane 2014; Sommerville et al. 2010; Songorwa 1999; Stone and Stone 2011; Stone and Nyaupane 2014; Stone and Rogerson 2011; Suich, 2013; Tantoh and Simatele 2017; Turpie and Letley 2021; Wainwright and Wehrmeyer 1998; Ward et al. 2017; Ward et al. 2018). For example, Sebele (2010) found that, in Botswana’s Khama Rhino Sanctuary, decision-making and benefit sharing were dominated by a small minority, leaving most community members excluded despite the land being communally owned. In the case of Avu Lagoon CREMA in Ghana, local elites used their positions of privilege to illicitly regain portions of the conservation land for private crop farming causing widespread disenfranchisement (Ahmed and Gasparatos 2020). Similarly, Oduor (2020) reported that institutional mechanisms for sharing benefits in Kenya’s Maasai Mara conservancies lacked transparency, accountability, and equity, and favoured individuals with political ties to conservancy leadership. In Zimbabwe, Shereni and Saarinen (2021) observed that communities living near Hwange National Park held negative views of the Communal Area Management Programme for Indigenous Resources (CAMPFIRE) project largely because the authorities retained most decision-making and management powers. In Madagascar, Ward et al. (2017) found that community participation in the management of Mangabe Forest was limited by miscommunication and lack of knowledge about who and how to get involved.

Some governance practices that favoured community participation were also observed. Leadership emerged as a central pillar of governance, with several case studies elaborating that charismatic individuals, elected representatives, and traditional authorities played pivotal roles in mobilising communities and sustaining participation (Abbot et al. 2001; Dyer et al. 2014; Silva and Mosimane 2014). Where collaboration between state agencies and local institutions was strong, governance frameworks were reinforced, enabling communities to better organise around conservation objectives, as was seen in the sacred groves of southwest Nigeria, the Zukpiri and Kunlog CREMAs and the Akwapim-Togo Mountain Range in Ghana, the Kamoa Sustainable Livelihoods Project in DR Congo, and marine conservation in Kigamboni, Kibiti, Kilwa, and Mafia districts in Tanzania (Adeyanju et al. 2022; Baddianaah and Baaweh 2021; Baker et al. 2018; Dyer et al. 2014; Katikiro et al. 2024; Robinson and Sasu 2013). The vital role played by conservation NGOs in facilitating local ownership and alleviating transaction costs, such as providing resources for community mobilisation and capacity development, cannot be overemphasised; even though external actors such as NGOs may not always prioritise local values (Abbot et al. 2001; Abebe et al. 2020; Ahmed and Gasparatos 2020; Baker et al. 2018; Balint and Mashinya 2006; Dyer et al. 2014; Jackson and Naughton-Treves 2012; Kachali and Loos 2025; Lepp and Holland 2006; Sommerville et al. 2010; Songorwa 1999). Inclusive engagement strategies empower marginalised groups such as women to participate, leading to broader community participation as was observed in conservancies such as Orupembe, Mashi, and King Nehale in Namibia (Lendelvo et al. 2012). In the Torra Conservancy also in Namibia, positive attitudes towards conservation were associated with devolution of substantial management authority to the local community which nurtured a sense of ownership (Scanlon and Kull 2009). In Ghana’s Wechiau Community Hippo Sanctuary, strong governance structures, anchored by a representative management board, ensured equitable distribution of benefits that spanned ethnic and gender divisions, strengthening cohesion and participation (Sheppard et al. 2010). Participatory governance processes that emphasised early community engagement were found to be effective in building foundations for long-term cooperation. Such processes underpinned the relative success of the Kamoa Sustainable Livelihoods Project in DR Congo (Dyer et al. 2014).

### Perceived Outcomes

The findings of this study show that both tangible (material) and intangible (non-material) outcomes play a critical role in determining people’s participation in conservation by conditioning perceptions of costs and benefits. Different types of natural resources (e.g. wildlife, forest, fisheries, rangelands, etc.) provide different combinations of incentives for participation that may be ecological, economic or social, depending on the uses and values attached to them by different resource users. Among these, economic incentives were particularly prominent in motivating participation, as shown across many case studies. For example, in a study of the Torra Conservancy in Namibia, 70% of respondents indicated that they did not support conservation before the introduction of direct benefits like monetary payments and game meat, even though they were already concerned about environmental degradation (Scanlon and Kull 2009). However, the findings also suggest that, in general, community participation in natural resources management is not driven by economic, social or ecological outcomes in isolation, but by their interaction and combined effect (Abbot et al. 2001; Adeyanju et al. 2022; Agyare et al. 2024; Ahmed and Gasparatos 2020; Carpenter, 2024; Drury O’Neill et al. 2024; Jackson and Naughton-Treves 2012; Lendelvo et al. 2012; Muyengwa 2015; Oburah et al. 2021; Oduor 2020; Robinson and Sasu 2013; Scanlon and Kull 2009; Silva and Mosimane 2014; Störmer et al. 2019; Tesfaye et al. 2012). For instance, while tourism income played a role in establishing the Kunlog CREMA in Ghana, the primary motivation was cultural, i.e. to protect bushbuck for future generations due to traditional taboos (Robinson and Sasu 2013). In a similar way, religio-cultural benefits were found to be the core driver of biodiversity conservation in the sacred groves of southwest Nigeria, even though economic incentives that emerged from tourism, job opportunities and income generation activities become major drivers of success (Adeyanju et al. 2022). In Namibia’s Mayuni Conservancy, community members remained committed to conservation despite unmet financial expectations, largely due to the social importance of being involved in community affairs (Silva and Mosimane 2014).

The reviewed case studies revealed a wide range of material and non-material benefits that motivated community participation in conservation to varying extents. Material benefits included financial income (e.g. tourism and hunting revenues, land lease payments, and payments for ecosystem services), business opportunities, financial assistance such as school bursaries and loan facilities, employment opportunities, enhanced food production (e.g. through agroforestry), enhanced access to resource (e.g. fuelwood, medicinal plants, edibles, non-timber forest products, grazing areas, game meat), improved social amenities and infrastructure (such as schools, healthcare facilities, roads, bridges, and water projects) and compensation for human-wildlife conflicts. Additional instrumental incentives that were cited are increased physical security (through monitoring and surveillance systems) and capacity-building opportunities (e.g. vocational and other kinds of trainings). Some case studies, especially concerning wildlife management, demonstrated that attitudes towards material benefits depended on the form in which they are received (e.g. individual or communal benefits and monetary or non-monetary benefits), their adequacy (e.g. in meeting opportunity costs), and the equitability of distribution (perceptions of fairness) (Carpenter, 2024; Muyengwa, 2015; Scanlon and Kull 2009; Störmer et al. 2019). Non-material motivators included community self-reliance, improved social relations, pride in environmental stewardship, fulfilment from participating in community affairs, and the reinforcement of traditional, cultural, spiritual, and aesthetic values (e.g. the intrinsic value of wildlife). Communities also valued improvements in natural resource conditions and provision of ecosystem services (Abbot et al. 2001; Adeyanju et al. 2022; Agyare et al. 2024; Ahmed and Gasparatos 2020; Akamani and Hall 2015; Baddianaah and Baaweh 2021; Bedelian et al. 2024; Carpenter, 2024; Dyer et al. 2014; Ezebilo, 2013; Hoole, 2010; Jackson and Naughton-Treves 2012; Katikiro et al. 2024; Lendelvo et al. 2012; Lepp and Holland 2006; Lewis and Phiri 1998; Mbaiwa, 2004; Meshack et al. 2006; Muyengwa, 2015; Oburah et al. 2021; Oduor, 2020; Robinson et al. 2021; Robinson and Sasu 2013; Scanlon and Kull 2009; Sebele, 2010; Sheppard et al. 2010; Shereni and Saarinen 2021; Silva and Mosimane 2014; Snyman, 2014; Sommerville et al. 2010; Stone and Rogerson 2011; Störmer et al. 2019; Suich, 2013; Tesfaye et al. 2012; Wainwright and Wehrmeyer 1998; Ward et al. 2017; Ward et al. 2018; Zewde et al. 2024).

The key perceived costs that discouraged community participation and, in some cases, led to acts of resistance that threatened the viability of the conservation initiatives included insufficient or absent economic benefits, high opportunity costs (costs of forgoing alternative land uses), burdensome transaction costs (costs of participating) and restricted access to natural resources. Other major disincentives were disempowerment from decision-making, unfair distribution of benefits, and frequent human-wildlife conflicts (Abebe et al. 2020; Ahmed and Gasparatos 2020; Bedelian et al. 2024; Carpenter, 2024; Dekker et al. 2020; Kachali and Loos 2025; Kahler et al. 2013; Kangalawe and Noe 2012; Kansky, 2022; Lendelvo et al. 2012; Lewis and Phiri 1998; Lubilo and Hebinck 2019; Mbaiwa, 2004; Meshack et al. 2006; Oduor, 2020; Rabe and Saunders 2013; Sebele 2010; Shereni and Saarinen 2021; Silva and Mosimane 2014; Snyman, 2014; Songorwa, 1999; Stone and Stone 2011; Stone and Rogerson 2011; Störmer et al. 2019; Suich, 2013; Tesfaye et al. 2012; Ward et al. 2017; Ward et al. 2018).

## Discussion

This study undertook a systematic review of peer-reviewed journal literature on collective management of environmental and natural resources in sub-Saharan Africa, with the aim of collating evidence on the key factors influencing community participation and cooperation. The reviewed articles overwhelmingly covered wildlife and forestry but also included other types of common-pool renewable natural resources such as marine resources, inland fisheries, rangelands, water resources, wetland, and watershed, drawing on case studies from 16 countries across the region. Through the application of an organizing framework, the study identified multiple interlinked factors and conditions underlying community participation that were grouped under six categories. These are: (i) individual-level characteristics which includes socio-economic traits such as gender, age and wealth, as well as individuals’ awareness and personal values; (ii) resource-specific attributes that entail the availability (accessibility) and ecological conditions of the natural resources under management, interceded by high resource dependence; (iii) community-level attributes particularly social cohesion and social capital that condition collective action; (iv) rule-related attributes including interaction between formal and informal rules, rule enforcement, rule legitimacy, and property rights; (v) process factors entailing governance and management processes and practices; and (vi) perceived outcomes which encompasses economic, social and environmental benefits and costs that are obtained or incurred.

Overall, the findings underscore the inherent complexity, multidimensionality, and interwoven nature of the factors shaping community participation. The evidence affirms that community participation (collective action) is highly context-specific, influenced by a dynamic interplay of historical, cultural, institutional, social, political and ecological conditions. Crucially, the findings support the consensus that local communities cannot be treated as monolithic entities; instead, they comprise individual members with diverse identities, interests, and capacities, whose potential motives (self-interest or co-operation) are activated by different social settings (e.g. Agrawal and Gibson 1999). As such, effective conservation planning should be grounded in comprehensive situational analyses that inform the development of adaptive, context-specific strategies that reflect the specific circumstances of each community, including its social heterogeneity. Such an approach would reduce the common tendency to apply overly simplified or generalized project design blue-prints that are often fundamentally flawed (Saunders, 2014). Informal environmental education and awareness creation on ecosystem services should also be an integral aspect of conservation planning because individuals’ knowledge and personal values strongly influence their attitudes and motivations to participate, impacting overall community participation. Environmental education can enrich value frameworks that underpin conservation behaviour. For example, it can accentuate local communities’ cultural attachment to land and wildlife that go beyond economic considerations. The contribution of conservation education to the success of community-based resource management approaches has been demonstrated by earlier reviews (Brooks 2017).

The review further highlights that different natural resource types such as forests, wildlife, rangelands, and fisheries, offer distinct configurations of environmental, economic, and social incentives that drive participation. While material or economic incentives undoubtedly play a significant role in motivating participation, especially owing to the high resource dependence which dictates provision of economic alternatives, the analysis reveals that participation in conservation is influenced as much by intrinsic and non-material motivations rooted in socio-cultural and environmental values, e.g. intrinsic value of wildlife. Consequently, conservation strategies that emphasis the reinforcement of existing socio-cultural and environmental values, alongside the provision of economic benefits (e.g. through payments for ecosystem services), are more likely to improve environmental stewardship in the long term. Similar conclusions have been reached by past systematic studies (Waylen et al. 2010). This is more so considering that, as demonstrated in case studies in this review, it remains difficult to distribute economic benefits equitable or manage them without causing disruptions to social dynamics. Furthermore, without strong governance, economic incentives linked to resource utilisation can lead to overexploitation rather than the conservation of resources (Hutton and Leader-Williams 2003; McNeely 1988; Rode et al. 2015). To this end, policy makers and conservation practitioners are well-advised to promote diversified and locally appropriate incentive structures that address the full spectrum of ecological, economic, and social motivations for participation, to ensure that important outcomes are not overlooked.

A consistent theme emerging from the literature is the central role of local governance structures and processes in collective action. Effective community participation is fundamentally contingent upon the strength, legitimacy, and functionality of local governance arrangements. Governance determines how decisions are made, who participates, how benefits and responsibilities are shared, and how conflicts are resolved. Many of the deep-rooted challenges identified across the reviewed case studies (e.g. marginalisation in decision-making and benefits access, and poor compliance with rules) are closely tied to weak or dysfunctional local governance systems, indicating a clear need to improve the governance of community participation interventions in general. This observation adds credence to the already existing evidence of the importance of strong institutions in facilitating collective action (Oldekop et al. 2010). Strengthening the governance of community-based approaches should entail not only improving the technical and administrative capacities of local institutions and organisations, but also embedding core principles of inclusivity, equity, transparency and accountability within their operational frameworks, including instituting accessible grievance redress systems. Importantly, institutional strengthening should pay greater attention to integrating informal institutions and traditional ecological knowledge with formal conservation frameworks to foster legitimacy and compliance. The functionality of local governance also depends on supportive national policies, legal recognition of land and resource rights, and linkages with regional and national institutions. Since NGOs and other development partners play a crucial role in facilitating local stewardship, particularly by providing requisite resources and expertise, it is vital that the agendas of local communities and such partners are mutually compatible, as also articulated in some past studies (Measham and Lumbasi 2013).

## Conclusion

In summary, this narrative review highlights that the underlying determinants of community participation are intricate and inextricably linked. It demonstrates that effective community engagement cannot be achieved through overly standardized approaches, as participation is shaped by diverse historical, cultural, socio-political, ecological, and institutional factors that are contextual. Recognizing the heterogeneity within communities is essential, as individual community members possess varying motivations and capacities to participate. The type of natural resource in question play a critical role in shaping participation, offering different bundles of ecological, economic, and social incentives. While material benefits are highly important, sustainable participation also depends on non-material or intrinsic outcomes. As such, policies and programs should be designed to support diverse, locally tailored incentive structures that mirror the full range of potential motivations. Finally, the quality of local governance institutions and organisations emerges as a decisive factor in shaping participation outcomes, which should be prioritised in conservation planning. The Institutional Analysis and Development framework enabled the exploration of multiple variables, including contextual and intrinsic factors, which influence resource use decisions in collective situations, hence it was practical and effective in guiding the study. While the study has provided a synthesis of the determinants of community participation in collective management of common-pool resources in sub-Saharan Africa, insights gained should be relevant to other developing countries where efforts are ongoing to translate community centred policies into practice, especially in cases with comparable social-ecological conditions such as high resource dependence and intra-community differentiations. Future research could explore the role of technological advances in facilitating community participation.

## Supplementary information


SUPPLEMENTARY MATERIAL 1


## Data Availability

No datasets were generated or analysed during the current study.
