# Supplementary material for: Determinants of Community Participation in Collective Management of Natural Resources in Sub-Saharan Africa: A Systematic Review
Source: Environ Manage. 2026 Aug 4;76(8):267. doi: 10.1007/s00267-026-02568-0 (PMC13437715; doi:10.1007/s00267-026-02568-0)
Supplement: Supplementary file 1 [file 267_2026_2568_MOESM1_ESM.pdf]

SUMMARY OF SELECTED ARTICLES FOR SYSTEMATIC REVEIW

No	Authors	Resource type	Model	Case Studies (Countries)	Subject of study	Activity	Suggested/ implemented interventions
1.	(Abbot et al., 2001)	Forest	ICDPs	Kilum-Ijim Forest (Cameroon)	Factors affecting attitudes and behaviours; success factors	Community forest management	Alternative livelihoods; trainings and capacity building; external support
2	(Abebe et al., 2020)	Wildlife	CBC	Bale Mountains (Ethiopia)	Perceptions of equity in distribution of benefits/ costs	Community Controlled hunting area	Transparent and inclusive procedure of decision making
3	(Adeyanju et al., 2022)	Forest	CBC	Osun-Osogbo; Idanre Hills; Igbo-Olodumare sacred groves (Nigeria)	Perceptions of socio-economic and religio-cultural benefits	Co-management of Sacred groves	Transparency in revenue sharing; collaboration between customary and formal government institutions
4	(Agyare et al., 2024)	Forest	CBNRM	Zukpiri; Murugu-Mognori; Asuo-Bia-Nkyirima; Aowin; River Asuopri; Achichire-Sureso-Pebaseman; Atewa; Weto CREMAs (Ghana)	Expectations and perceived outcomes	Community Resource Management Areas (CREMAs)	External support; diversifying incentive structure
5	(Ahmed & Gasparatos, 2020)	Floodplain	CBNRM	Avu Lagoon CREMA (Ghana)	Land politics	CREMA	Compensation for lost access
6	(Akamani & Hall, 2015)	Forest	CM	Kwapanin; Kyirayaso communities (Ghana)	Determinants of participation	Collaborative forest management	Promote equitable resilience
7	(Baddianaah & Baaweh, 2021)	Forest	CBNRM	Zukpiri CREMA (Ghana)	Challenges	CREMA	Explore livelihood alignment programmes
8	(Baker et al., 2018)	Forest	CBNRM	Akwapim-Togo mountain range; Zukpiri CREMA (Ghana)	Challenges	CREMA	Improving linkages between customary and state

SUMMARY OF SELECTED ARTICLES FOR SYSTEMATIC REVEIW

							institutions; capacity building and training
9	(Baker-Médard et al., 2021)	Marine	CBC	19 fishing villages with marine reserves (Madagascar)	Social-economic drivers of participation	Marine conservation and fisheries	Inclusive representation
10	(Balint & Mashinya, 2006)	Wildlife	CBNRM/ CBC	Mahenye CAMPFIRE, (Zimbabwe)	Factors affecting performance	CAMPFIRE	External support; capacity building
11	(Bedelian et al., 2024)	Wildlife	CBC	Naboisho; Mara North; Olare-Motorogi conservancies (Kenya)	Determinants of participation	Wildlife conservation and tourism	Consideration of contextual and distributive equity
12	(Carpenter, 2024)	Wildlife	CBC	Puros; Sesfontein; Anabeb; Omatendeka conservancies (Namibia)	Effect of benefits and costs	Wildlife Conservation	Cultural considerations
13	(Cerbu et al., 2013)	Forest	ICDPs	Akok; Awae communities (Cameroon)	Community capacities	REDD+	Capacity-building; reinforcing livelihood strategies that minimize forest loss.
14	(Dekker et al., 2020)	Rangelands	CBNRM	Enduimet WMA (Tanzania)	Conflicting rationalities	Pasture management	Recognition power and inequality
15	(Drury O'Neill et al., 2024)	Marine	CM	3 villages in Unguja and Pemba (Tanzania)	Motivations for compliance	Co-management of marine fisheries	Providing discussion spaces for conservation participants
16	(Dyer et al., 2014)	Forest	CBNRM	Katanino JFM (Zambia); Kamoa SL Program (DR Congo); Nhambita Community Carbon Project (Mozambique)	Process based factors	Joint forest management; conservation agriculture; tree-planting for carbon	Empowerment; two-way communication; charismatic leadership
17	(Etiegni et al., 2017)	Fisheries	CM	4 Beach Management Units in Lake Victoria (Kenya)	Role of Institutions	Fisheries co-management	Re-design of co-management structures

SUMMARY OF SELECTED ARTICLES FOR SYSTEMATIC REVEIW

18	(Ezebilo, 2013)	Forest	ICDPs	Okwango Division of Cross River National Park (Nigeria)	Incentives	Forest conservation	Capacity-building; strict monitoring and control
19.	(Hoole, 2010)	Wildlife	CBNRM	Torra; Ehi-rovipuka conservancies (Namibia)	Partnerships	Ecotourism	Equitable power sharing with communities
20	(Jackson & Naughton-Treves, 2012)	Forest	ICDPs	Arabuko-Sokoke (Kenya)	Incentives, attitudes and perceptions	PES	Hybrid approach between PES and ICDPs
21	(Kachali & Loos, 2025)	Wildlife	CBNRM	Mukungule; Munyamadzi; Musalangu GMAs (Zambia)	Perceptions of equity	Game Management Area (GMA)	Training for NGO and government actors on local worldviews; decentralize HWC intervention decision-making; autonomous mediation forums
22	(Kahler et al., 2013)	Wildlife	CBNRM	Wuparo; Dzoti conservancies (Namibia)	Stakeholder perception on poaching, wildlife damage, benefits	Wildlife conservation	Multiple strategies to reduce poaching
23.	(Kaluma & Umar, 2021)	Fisheries	CM	Mweru- Luapula fishery (Zambia)	Participation and compliance	Fisheries co-management	A common property regime with locally designed operational rules
24	(Kangalawe & Noe, 2012)	Forest/ wildlife	CBC	Mbarang'andu WMA (Tanzania)	Poverty alleviation	Wildlife Management Area (WMA)	Reconsider resource access mechanisms
25	(Kansky, 2022)	Wildlife	CBNRM	Bamunu; Balyerwa; Mayuni conservancies (Namibia)	Human wildlife conflict	Wildlife conservation	Improve information flow; an adaptive management mitigation measure program.
26	(Katikiro et al., 2024)	Marine	CBNRM/ CBC	Kigamboni; Kibiti; Kilwa; and Mafia districts (Tanzania)	Enabling conditions	Fisheries	Innovative

							financing schemes and private sector partnerships
27	(Katikiro et al., 2015)	Marine	CBNRM	Mnazi Bay-Ruvuma Estuary Marine Park (Tanzania)	Challenges	Marine conservation	Enhanced community involvement at initial stages (endogenous approach)
28	(Lendelvo et al., 2012)	Wildlife/ rangelands	CBNRM	Orupembe; Mashi; Torra; King Nehale conservancies (Namibia)	Women's participation	Wildlife conservation/ rangelands management	Women participation in their traditional roles
29	(Lepp & Holland, 2006)	Wetlands	CBNRM	Bigodi's Magombe Swamp- KAFRED (Uganda)	Attitudes	Conservation and ecotourism	Inclusion of local people
30.	(Lewis & Phiri, 1998)	Wildlife	CBC	Luangwa Integrated Rural Development Project- (Zambia)	Benefits and behaviour change	GMA	Well defined legal rights that guarantee entitled revenue shares; well defined responsibilities for managing resource by the community supported by capacity-building rather than by external management.
31	(Lubilo & Hebinck, 2019)	Wildlife	CBNRM/ CBC	Wuparo, Sobbe and Kwandu conservancies (Namibia)	Hunting behaviour	Wildlife conservation	Review policy strategies and approaches to ensure various interests groups are integrated
32.	(Mbaiwa, 2004)	Wildlife	CBNRM	Okavango Community Trust; Okavango Kopano Mokoro Community Trust; Khwai Development Trust (Botswana)	Outcomes and challenges	Wildlife conservation	Training and capacity building

33	(Meshack et al., 2006)	Forest	CBNRM	Ambangulu Forest (Tanzania)	Costs and benefits	Joint forest management	Sharing conservation costs among stakeholders
34	(Moyo et al., 2017)	Wildlife	CBC	Burunge Wildlife Management Area (Tanzania)	Resource access	WMA	Learn from and incorporate local experiences
35	(Muyengwa, 2015)	Wildlife	CBNRM	Balyerwa; Kwandu; Mashi; Sobbe; Wuparo conservancies (Namibia)	Incentives	Wildlife conservation	Pay attention to the types of incentives, participation mechanisms and community level factors
36	(Oburah et al., 2021)	Wildlife	CBC	Naibunga conservancy (Kenya)	Perceptions and involvement	Wildlife conservation	Maximizing socioeconomic benefits for local pastoralists
37	(Oduor, 2020)	Wildlife	CBNRM	Mara North; Olare-Motorogi, Lemek; Naboisho; Olderkesi conservancies (Kenya)	Livelihood impacts and governance processes	Wildlife conservation and ecotourism	Strengthen governance institutions
38	(Rabe & Saunders, 2013)	Forest	CBNRM	Jozani-Pete, Tanzania	Decision-making	Mangrove conservation	Address existing power relations and deliver on promises of material benefits
39	(Robinson et al., 2021)	Rangelands	CBNRM	Shompole; Olkiramatian; Il'Ngwesi conservancies (Kenya), and Naniga-Dera; Dirre (Ethiopia)	Challenges	Participatory range management	Multi-level approach to participatory rangeland management
40	(Robinson & Sasu, 2013)	Wildlife	CBNRM	Kunlog CREMA (Ghana)	Conservation values	CREMA	Material benefits should not be the sole focus
41	(Saunders et al., 2008)	Forest	CBNRM	Kisakasaka (Tanzania)	Governance arrangements	Mangrove conservation	Reintegration with village level decision-making structures
42	(Scanlon & Kull, 2009)	Wildlife	CBNRM	Torra Conservancy (Namibia)	Benefits and attitudes	Conservation	Material benefits are specific, equitable,

							appropriate and sufficient; empower representative local institutions; build on intangibles benefits
43	(Sebele, 2010)	Wildlife	CBNRM	Khama Rhino Sanctuary Trust, (Botswana)	Benefits and challenges	Conservation and tourism	Enabling policies
44	(Sheppard et al., 2010)	Wildlife	CBNRM	Wechiau Community Hippo Sanctuary (Ghana)	Outcomes	Wildlife conservation and ecotourism	learn about the community's spiritual, cultural and economic connections to nature.
45	(Shereni & Saarinen, 2021)	Wildlife	CBNRM	Hwange National Park, (Zimbabwe)	Perceptions	CAMPFIRE	Revive village committees; HWC mitigation
46	(Silva & Mosimane, 2014)	Wildlife	CBNRM	Uibasen; Mayuni conservancies, (Namibia)	Economic and social motivations	Wildlife conservation	Prioritizing social cohesion
47	(Snyman, 2014)	Wildlife	CBC	Okavango Community Trust (Botswana); Liwonde National Park (Malawi); Okondjombo; Purros; Sanitatas; Orupembe; Torra; Anabeb; Sesfontein conservancies (Namibia); Mpukane; Makuleke communities (S.Africa); Malama (Zambia); Tsholotsho district (Zimbabwe)	Attitudes	Conservation and ecotourism	Consideration of demographic and socio-economic factors
48	(Sommerville et al., 2010)	Forest	CBC	Menabe community-based PES (Madagascar)	Fairness and benefits distribution	PES	Monitoring social indicators
49	(Songorwa, 1999)	Wildlife	CBC	Selous Conservation Programme (Tanzania)	Community interest	WMA	Raise genuine interest in wildlife conservation among community members.

SUMMARY OF SELECTED ARTICLES FOR SYSTEMATIC REVEIW

50	(Stone & Stone, 2011)	Wildlife	CBNRM	Khama Rhino Sanctuary Trust (Botswana)	Challenges	Conservation	Awareness creation; dialogue between stakeholders
51	(Stone & Nyaupane, 2014)	Wildlife	CBNRM	Nata Bird Sanctuary (Botswana)	Community differentiations	Conservation and ecotourism	Recognise local heterogeneities
52	(Stone & Rogerson, 2011)	Wildlife	CBNRM	Nata Bird Sanctuary (Botswana)	Perceptions of benefits	Ecotourism	Community involvement in planning and implementation
53	(Störmer et al., 2019)	Wildlife	CBNRM	18 conservancies in Erongo-Kunene and Zambezi (Namibia)	Attitudes	Wildlife conservation	Addressing both tangible and intangible benefits
54	(Suich, 2013)	Wildlife	CBNRM	Tchuma Tchato project in Tete, (Mozambique); Kwandu Conservancy (Namibia)	Economic incentives	Wildlife conservation	Provide appropriate incentives
55	(Tantoh & Simatele, 2017)	Water resources	CBNRM	Tugi; Zang-Tabi; Baicham; Muloin; Njimkang; Ngarum villages (Cameroon)	Challenges	Water resources management	Ensures local people are empowered, integrated and encouraged to participate
56	(Tesfaye et al., 2012)	Forest	PM	Dodola forest (Ethiopia)	Attitudes	Participatory forest management	Continuous exchange of ideas and information between user groups and the government
57	(Turpie & Letley, 2021)	Wildlife	CBNRM	Sorris-Sorris; Tsiseb; Uibasen-Twyfelfontein conservancies, (Namibia)	Trust, factors influencing co-operation	Wildlife conservation	External oversight
58	(Wainwright & Wehrmeyer, 1998)	Wildlife	CBNRM	Luangwa Integrated Resource Development Project (Zambia)	Successes and failures	Wildlife conservation	Capacity-building and conservation education; creation of dynamic women's program

59	(Ward et al., 2017)	Forest	CM	Mangabe Forest (Madagascar)	Barriers and drivers	Protected area co-management	Understanding the heterogeneous nature of communities; ensuring all households are represented in governance participation
60	(Ward et al., 2018)	Forest	CM	Mangabe Forest (Madagascar)	Livelihood impacts	Protected area co-management	Holistic view of local livelihoods
61	(Zewde et al., 2024)	Watershed	PM	Jemma Watershed (Ethiopia)	Perceptions and attitudes	Integrated watershed management	Creating public awareness; formulate regulations to ensure equitable benefit-sharing; develop two-way communication system; integrate indigenous knowledge

Abbot, J. I. O., Thomas, D. H. L., Gardner, A. A., Neba, S. E., & Khen, M. W. (2001). Understanding the links between conservation and development in the Bamenda Highlands, Cameroon. *World Development, 29*(7), 1115-1136. doi:10.1016/S0305-750X(01)00033-X

Abebe, B. A., Jones, K. W., Solomon, J., Galvin, K., & Evangelista, P. (2020). Examining social equity in community-based conservation programs: A case study of controlled hunting programs in Bale Mountains, Ethiopia. *World Development, 135*. doi:10.1016/j.worlddev.2020.105066

Adeyanju, S. O., Bulkan, J., Onyekwelu, J. C., St-Laurent, G. P., Kozak, R., Sunderland, T., & Stimm, B. (2022). Drivers of Biodiversity Conservation in Sacred Groves: A Comparative Study of Three Sacred Groves in Southwest Nigeria. *International Journal of the Commons, 16*(1), 94-107. doi:10.5334/ijc.1143

Agyare, A. K., Holbech, L. H., & Arcilla, N. (2024). Great expectations, not-so-great performance: Participant views of community-based natural resource management in Ghana, West Africa. *Current Research in Environmental Sustainability, 7*. doi:10.1016/j.crsust.2024.100251

Ahmed, A., & Gasparatos, A. (2020). Reconfiguration of land politics in community resource management areas in Ghana: Insights from the Avu Lagoon CREMA. *Land Use Policy, 97*, 104786.

Akamani, K., & Hall, T. E. (2015). Determinants of the process and outcomes of household participation in collaborative forest management in Ghana: A quantitative test of a community resilience model. *Journal of Environmental Management, 147*, 1-11.

SUMMARY OF SELECTED ARTICLES FOR SYSTEMATIC REVEIW

Baddianaah, I., & Baaweh, L. (2021). The Prospects Of Community-Based Natural Resource Management In Ghana: A Case Study Of Zukpiri Community Resource Management Area. *Heliyon, 7*(10). doi:10.1016/j.heliyon.2021.e08187

Baker-Médard, M., Gantt, C., & White, E. R. (2021). Classed conservation: Socio-economic drivers of participation in marine resource management. *Environmental Science and Policy, 124*, 156-162. doi:10.1016/j.envsci.2021.06.007

Baker, D. M., Murray, G., & Agyare, A. K. (2018). Governance and the making and breaking of social-ecological traps. *Ecology and Society, 23*(1). doi:10.5751/ES-09992-230138

Balint, P. J., & Mashinya, J. (2006). The decline of a model community-based conservation project: Governance, capacity, and devolution in Mahenye, Zimbabwe. *Geoforum, 37*(5), 805-815. doi:10.1016/j.geoforum.2005.01.011

Bedelian, C., Ogutu, J. O., Homewood, K., & Keane, A. (2024). Evaluating the determinants of participation in conservancy land leases and its impacts on household wealth in the Maasai Mara, Kenya: Equity and gender implications. *World Development, 174*, 106442.

Carpenter, S. (2024). The varying effects of benefit types on community members' views of whether they gain from wildlife. *Conservation Science and Practice, 6*(9). doi:10.1111/csp2.13217

Cerbu, G. A., Sonwa, D. J., & Pokorny, B. (2013). Opportunities for and capacity barriers to the implementation of REDD+ projects with smallholder farmers: Case study of Awae and Akok, Centre and South Regions, Cameroon. *Forest Policy and Economics, 36*, 60-70. doi:10.1016/j.forpol.2013.06.018

Dekker, L. A., Arts, K., & Turnhout, E. (2020). From rationalities to practices: Understanding unintended consequences of CBNRM. *Conservation and Society, 18*(2), 137-147.

Drury O'Neill, E., Daw, T. M., Mwaipopo, R. N., & Lindkvist, E. (2024). The complexity of compliance—Diverse responses to octopus fishery closures in Zanzibar. *People and Nature, 6*(6), 2543-2563. doi:10.1002/pan3.10742

Dyer, J., Stringer, L. C., Dougill, A. J., Leventon, J., Nshimbi, M., Chama, F., . . . Syampungani, S. (2014). Assessing participatory practices in community-based natural resource management: Experiences in community engagement from southern Africa. *Journal of Environmental Management, 137*, 137-145. doi:10.1016/j.jenvman.2013.11.057

Etiegni, C., Irvine, K., & Kooy, M. (2017). Playing by whose rules? Community norms and fisheries rules in selected beaches within Lake Victoria (Kenya) co-management. *Environment, Development and Sustainability, 19*, 1557-1575.

Ezebilo, E. E. (2013). Nature conservation in Cross River National Park, south-east Nigeria: promoting collaboration between local people and conservation authorities. *International Journal of Biodiversity Science, Ecosystem Services & Management, 9*(3), 215-224.

Hoole, A. F. (2010). Place-power-prognosis: Community-based conservation, partnerships, and ecotourism enterprises in Namibia. *International Journal of the Commons, 4*(1).

Jackson, M. M., & Naughton-Treves, L. (2012). Eco-bursaries as incentives for conservation around Arabuko-Sokoke Forest, Kenya. *Environmental Conservation, 39*(4), 347-356. doi:10.1017/S0376892912000161

Kachali, R. N., & Loos, J. (2025). Unveiling disparities between planned and perceived equity arrangements in protected area co-governance: Evidence from the North Luangwa Ecosystem in Zambia. *Environmental Science and Policy, 169*. doi:10.1016/j.envsci.2025.104068

Kahler, J. S., Roloff, G. J., & Gore, M. L. (2013). Poaching Risks in Community-Based Natural Resource Management. *Conservation Biology, 27*(1), 177-186. doi:10.1111/j.1523-1739.2012.01960.x

SUMMARY OF SELECTED ARTICLES FOR SYSTEMATIC REVEIW

Kaluma, K., & Umar, B. B. (2021). Outcomes of participatory fisheries management: An example from co-management in Zambia's Mweru-Luapula fishery. *Heliyon, 7*(2).

Kangalawe, R. Y., & Noe, C. (2012). Biodiversity conservation and poverty alleviation in Namtumbo District, Tanzania. *Agriculture, ecosystems & environment, 162*, 90-100.

Kansky, R. (2022). Unpacking the challenges of wildlife governance in community-based conservation programs to promote human–wildlife coexistence. *Conservation Science and Practice, 4*(10). doi:10.1111/csp2.12791

Katikiro, R., Mahajan, S. L., & Medard, M. (2024). The establishment, persistence, and scaling of marine community-based natural resource management in four coastal districts in Tanzania. *Marine Policy, 169*. doi:10.1016/j.marpol.2024.106341

Katikiro, R. E., Macusi, E. D., & Ashoka Deepananda, K. H. M. (2015). Challenges facing local communities in Tanzania in realising locally-managed marine areas. *Marine Policy, 51*, 220-229. doi:10.1016/j.marpol.2014.08.004

Lendelvo, S., Munyebvu, F., & Suich, H. (2012). Linking women's participation and benefits within the Namibian community based natural resource management program. *Journal of Sustainable Development, 5*(12), 27.

Lepp, A., & Holland, S. (2006). A comparison of attitudes toward state-led conservation and community-based conservation in the village of Bigodi, Uganda. *Society and Natural Resources, 19*(7), 609-623.

Lewis, D. M., & Phiri, A. (1998). Wildlife snaring–an indicator of community response to a community-based conservation project. *ORYX, 32*(2), 111-121.

Lubilo, R., & Hebinck, P. (2019). 'Local hunting' and community-based natural resource management in Namibia: Contestations and livelihoods. *Geoforum, 101*, 62-75. doi:10.1016/j.geoforum.2019.02.020

Mbaiwa, J. E. (2004). The success and sustainability of community-based natural resource management in the okavango delta, botswana. *South African Geographical Journal, 86*(1), 44-53. doi:10.1080/03736245.2004.9713807

Meshack, C. K., Ahdikari, B., Doggart, N., & Lovett, J. C. (2006). Transaction costs of community-based forest management: Empirical evidence from Tanzania. *African Journal of Ecology, 44*(4), 468-477. doi:10.1111/j.1365-2028.2006.00659.x

Moyo, F., Funk, S., & Pretzsch, J. (2017). Between policy intent and practice: Negotiating access to land and other resources in Tanzania's wildlife management areas. *Tropical Conservation Science, 10*, 1940082917744167.

Muyengwa, S. (2015). Determinants of individual level satisfaction with community based natural resources management: A case of five communities in Namibia. *Environments - MDPI, 2*(4), 608-623. doi:10.3390/environments2040608

Oburah, K. O., Lenachuru, C., & Odadi, W. O. (2021). Does the community conservancy model work for pastoralists? Insights from Naibunga conservancy in Northern Kenya. *Sustainability (Switzerland), 13*(14). doi:10.3390/su13147772

Oduor, A. M. (2020). Livelihood impacts and governance processes of community-based wildlife conservation in Maasai Mara ecosystem, Kenya. *Journal of Environmental Management, 260*, 110133.

Rabe, L., & Saunders, F. (2013). Community-based Natural Resource Management of the Jozani-Pete Mangrove Forest: Do they Have a voice? *Western Indian Ocean Journal of Marine Science, 12*(2), 133-150.

Robinson, L. W., Eba, B., Flintan, F., Frija, A., Nganga, I. N., Ontiri, E. M., . . . Moiko, S. S. (2021). The Challenges of Community-Based Natural Resource Management in Pastoral Rangelands. *Society and Natural Resources, 34*(9), 1213-1231. doi:10.1080/08941920.2021.1946629

SUMMARY OF SELECTED ARTICLES FOR SYSTEMATIC REVEIW

Robinson, L. W., & Sasu, K. A. (2013). The role of values in a community-based conservation initiative in northern Ghana. *Environmental Values, 22*(5), 647-664. doi:10.3197/096327113X13745164553914

Saunders, F., Mohammed, S. M., Jiddawi, N., & Sjöling, S. (2008). An examination of governance arrangements at Kisakasaka Mangrove Reserve in Zanzibar. *Environmental Management, 41*(5), 663-675. doi:10.1007/s00267-007-9050-x

Scanlon, L., & Kull, C. A. (2009). Untangling the links between wildlife benefits and community-based conservation at Torra Conservancy, Namibia. *Development Southern Africa, 26*(1), 75-93. doi:10.1080/03768350802640107

Sebele, L. S. (2010). Community-based tourism ventures, benefits and challenges: Khama Rhino Sanctuary Trust, Central District, Botswana. *Tourism Management, 31*(1), 136-146. doi:10.1016/j.tourman.2009.01.005

Sheppard, D. J., Moehrenschlager, A., McPherson, J. M., & Mason, J. J. (2010). Ten years of adaptive community-governed conservation: evaluating biodiversity protection and poverty alleviation in a West African hippopotamus reserve. *Environmental Conservation, 37*(3), 270-282.

Shereni, N. C., & Saarinen, J. (2021). Community perceptions on the benefits and challenges of community-based natural resources management in Zimbabwe. *Development Southern Africa, 38*(6), 879-895. doi:10.1080/0376835X.2020.1796599

Silva, J. A., & Mosimane, A. (2014). "How Could I Live Here and Not Be a Member?": Economic Versus Social Drivers of Participation in Namibian Conservation Programs. *Human Ecology, 42*(2), 183-197. doi:10.1007/s10745-014-9645-9

Snyman, S. (2014). Assessment of the main factors impacting community members' attitudes towards tourism and protected areas in six southern African countries. *Koedoe, 56*(2). doi:10.4102/koedoe.v56i2.1139

Sommerville, M., Jones, J. P. G., Rahajaharison, M., & Milner-Gulland, E. J. (2010). The role of fairness and benefit distribution in community-based Payment for Environmental Services interventions: A case study from Menabe, Madagascar. *Ecological Economics, 69*(6), 1262-1271. doi:10.1016/j.ecolecon.2009.11.005

Songorwa, A. N. (1999). Community-based wildlife management (CWM) in Tanzania: are the communities interested? *World Development, 27*(12), 2061-2079.

Stone, L. S., & Stone, T. M. (2011). Community-based tourism enterprises: Challenges and prospects for community participation; Khama Rhino Sanctuary trust, Botswana. *Journal of Sustainable Tourism, 19*(1), 97-114. doi:10.1080/09669582.2010.508527

Stone, M. T., & Nyaupane, G. (2014). Rethinking community in community-based natural resource management. *Community Development, 45*(1), 17-31. doi:10.1080/15575330.2013.844192

Stone, M. T., & Rogerson, C. M. (2011). Community-based natural resource management and tourism: Nata bird sanctuary, Botswana. *Tourism Review International, 15*(1-2), 159-169.

Störmer, N., Weaver, L. C., Stuart-Hill, G., Diggle, R. W., & Naidoo, R. (2019). Investigating the effects of community-based conservation on attitudes towards wildlife in Namibia. *Biological Conservation, 233*, 193-200. doi:10.1016/j.biocon.2019.02.033

Suich, H. (2013). The effectiveness of economic incentives for sustaining community based natural resource management. *Land Use Policy, 31*, 441-449. doi:10.1016/j.landusepol.2012.08.008

Tantoh, H. B., & Simatele, D. (2017). Community-based water resource management in North-west Cameroon: the role of potable water supply in community development. *South African Geographical Journal, 99*(2), 166-183. doi:10.1080/03736245.2016.1208589

SUMMARY OF SELECTED ARTICLES FOR SYSTEMATIC REVEIW

Tesfaye, Y., Roos, A., & Bohlin, F. (2012). Attitudes of local people towards collective action for forest management: The case of participatory forest management in Dodola area in the Bale Mountains, Southern Ethiopia. *Biodiversity and Conservation, 21*, 245-265.

Turpie, J., & Letley, G. (2021). Would community conservation initiatives benefit from external financial oversight? A framed field experiment in Namibia's communal conservancies. *World Development, 142*. doi:10.1016/j.worlddev.2021.105442

Wainwright, C., & Wehrmeyer, W. (1998). Success in integrating conservation and development? A study from Zambia. *World Development, 26*(6), 933-944.

Ward, C., Holmes, G., & Stringer, L. (2017). Perceived barriers to and drivers of community participation in protected-area governance. *Conservation Biology, 32*(2), 437-446. doi:10.1111/cobi.13000

Ward, C., Stringer, L. C., & Holmes, G. (2018). Protected area co-management and perceived livelihood impacts. *Journal of Environmental Management, 228*, 1-12. doi:10.1016/j.jenvman.2018.09.018

Zewde, N. T., Denboba, M. A., & Tadesse, S. A. (2024). Perceptions and attitudes of local people towards participatory natural resources management in the Jemma Watershed, North Shewa Zone, Ethiopia. *Sustainable Environment, 10*(1). doi:10.1080/27658511.2024.2310400
